# Measuring motivational relationship processes in experience sampling: A reliability model for moments, days, and persons nested in couples

**DOI:** 10.3758/s13428-021-01701-7

**Published:** 2021-11-01

**Authors:** Felix D. Schönbrodt, Caroline Zygar-Hoffmann, Steffen Nestler, Sebastian Pusch, Birk Hagemeyer

**Affiliations:** 1 Department of Psychology, Ludwig-Maximilians-Universität München, Leopoldstr. 13, 80802 München, Germany; 2 University of Münster, Münster, Germany; 3 Friedrich Schiller University Jena, Jena, Germany

**Keywords:** Relationship, Motivation, Intensive longitudinal designs, Change reliability, Experience sampling, Ambulatory assessment

## Abstract

The investigation of within-person process models, often done in experience sampling designs, requires a reliable assessment of within-person change. In this paper, we focus on dyadic intensive longitudinal designs where both partners of a couple are assessed multiple times each day across several days. We introduce a statistical model for variance decomposition based on generalizability theory (extending P. E. Shrout & S. P. Lane, 2012), which can estimate the relative proportion of variability on four hierarchical levels: moments within a day, days, persons, and couples. Based on these variance estimates, four reliability coefficients are derived: between-couples, between-persons, within-persons/between-days, and within-persons/between-moments. We apply the model to two dyadic intensive experience sampling studies (*n*_1_ = 130 persons, 5 surveys each day for 14 days, ≥ 7508 unique surveys; *n*_2_ = 508 persons, 5 surveys each day for 28 days, ≥ 47764 unique surveys). Five different scales in the domain of motivational processes and relationship quality were assessed with 2 to 5 items: State relationship satisfaction, communal motivation, and agentic motivation; the latter consists of two subscales, namely power and independence motivation. Largest variance components were on the level of persons, moments, couples, and days, where within-day variance was generally larger than between-day variance. Reliabilities ranged from .32 to .76 (couple level), .93 to .98 (person level), .61 to .88 (day level), and .28 to .72 (moment level). Scale intercorrelations reveal differential structures between and within persons, which has consequences for theory building and statistical modeling.

Variability is an inherent aspect of virtually all conceptualizations of the term motivation (e.g., Berridge, 2004; McClelland, 1987). Our momentary wishes and desires not only depend on past experiences (e.g., we get hungry when we have not eaten for some time), but also on situational cues, that signal the current availability of incentives, and the presence of competing desires. In motivation research, situational factors are usually manipulated in laboratory experiments to test causal hypotheses concerning the conditions and consequences of motivational states (Heckhausen & Heckhausen, 2018; Schultheiss & Brunstein, 2010; Schultheiss & Köllner, 2021).

However, experimental studies tell us little about the time scale on which motivational states vary in everyday life. Is motivation waxing and waning from moment to moment within a day? Or is it a rather slow process that ramps up over several days, with little within-day fluctuation? Does it follow a weekly rhythm with some desires being stronger on weekends and weaker on workdays? Beyond these different time scales, motivational states might also vary between persons (Fleeson, 2001), which is a core assumption underlying research on motive dispositions (Hagemeyer, Neyer, Neberich, & Asendorpf, 2013; Schönbrodt & Gerstenberg, 2012; Schultheiss & Köllner, 2021). In addition, couples or even larger groups of people could be distinguishable in terms of their typical motivation, which adds additional potential levels of variability.

In our analyses of the time scale and levels of variability of several motivational constructs, we extend an existing statistical model for variance decomposition and reliability estimation (Cranford et al., 2006; Shrout & Lane, 2012) with an additional temporal level (moments within a day) and dyadic interdependence. For such statistical analyses, intensive longitudinal assessments of people’s motivational states as they occur in their everyday lives are necessary (i.e., experience sampling studies; Hofmann, Finkel, & Fitzsimons, 2015; Hofmann, Vohs, & Baumeister, 2012; Zygar et al.,, 2018b; Bolger & Laurenceau, 2013; Laurenceau & Bolger, 2005). In this study, we focus on the dynamics of motivation in the life-domain of romantic relationships. Specifically, we investigate the variability and reliability of self-reported communal and agentic motivational states and relationship satisfaction as assessed in two intensive experience sampling studies. For this purpose, we propose a model for variance decomposition and reliability estimation that covers an ESM data structure where the order of multiple moments is crossed with days, days are crossed with persons, and persons are nested in couples.

Knowledge about the time scale and variability of motivational processes carries important information for the design of studies. For example, the frequency and time points of momentary assessment should match the time scale of variability, and limited resources call for a trade-off analysis whether short and intensive (within day) measurements, or longer (but less intensive) daily diaries, are more appropriate for the research question at hand. Furthermore, scale correlations on the between-person level usually do not reflect within-person processes (Molenaar, 2008). However, often within-person conclusions are drawn from between-person studies, which can result in an ecological fallacy such as the Simpson’s paradox (Adolf & Fried, 2019; Medaglia, Jeronimus, & Fisher, 2019; Fisher, Medaglia, & Jeronimus, 2018; Kievit, Frankenhuis, Waldorp, & Borsboom, 2013). Consequently, scale intercorrelations can differ depending on the level of analysis. Just as reliability has to be considered on each level of analysis, construct validity also has to be analyzed on each level (Shrout & Lane, 2012; Horstmann & Ziegler, 2020).

In selecting motivational variables relevant for romantic relationships, we relied on the conceptualization of partner-related agentic and communal motives, as proposed by Hagemeyer and Neyer (2012). According to this view, agentic motivations focus on the individual self and strivings for independence and power in the relationship. Although independence and power are distinguishable classes of goals, both facets have in common that they entail a sense of psychological distance from one’s relationship partner. In terms of the hierarchy in a romantic relationship, independence strivings can be viewed as providing horizontal distance to one’s partner, whereas power strivings provide vertical distance. Thus, independence and power are related to different behavioral strategies of motive implementation (independence strivings often lead to physical distance from the partner, whilst power might often be exerted in close proximity), but they share a common incentive, namely the experience of feeling as a capable and self-reliant individual. Communal strivings, on the other hand, are directed towards experiences of closeness and community with one’s partner. According to Hagemeyer and Neyer (2012), they manifest in “enjoying joint activities and closeness, sharing of experiences and resources, sympathetic concern, efforts to improve the relationship, and feelings of loneliness in absence of the partner” (p. 116). These definitions were derived from Bakan’s (1966) original concepts of agency and communion, and, accordingly, they are viewed as fundamental motivational dimensions in romantic relationships (Hagemeyer & Neyer, 2012; Hagemeyer, Neyer, Neberich, & Asendorpf, 2013). Previous studies mainly focused on partner-related agency and communion at the between-person level of motive dispositions and largely confirmed expected associations between the motives and measures of relationship quality (Hagemeyer, Neberich, Asendorpf, & Neyer, 2013; Hagemeyer & Neyer, 2012; Hagemeyer et al.,, 2013; Hagemeyer, Schönbrodt, Neyer, Neberich, & Asendorpf, 2015). Overall, self-reported (explicit) and indirectly assessed (implicit) agency motives showed negative associations, whereas communal motives showed positive associations with relationship quality.

There is increasing interest in the analysis of daily motivational processes in couples, for example focusing on helping motivation (Kindt, Vansteenkiste, Loeys, & Goubert, 2016), motives for sacrifice in intimate relationships (Impett, Gable, & Peplau, 2005), or sexual motivation (Muise, Impett, & Desmarais, 2013; Dewitte & Mayer, 2018). Concerning our focal constructs, we are only aware of three previous studies that addressed partner-related communion and agency motivation within partners of a couple in a longitudinal design. Hagemeyer, Schönbrodt, Neyer, Neberich, and Asendorpf (2015), Study 2, found in a two-week daily diary of 106 couples that daily relationship satisfaction in general was increased when partners spent more time together. However this positive effect of physical proximity was diminished in coresiding couples, when partners reported high state agency motivation. Further, in experience-sampling design with five assessments per day, momentary variations in self-reported partner-related communal and agentic motivation (over the course of a few hours) were positively related to variations in communal and agentic behavior, which corresponds to findings on the between-person level (Zygar et al.,, 2018b; Zygar-Hoffmann, Pusch, Hagemeyer, & Schönbrodt, 2020), and communal motivation was predictive of relationship satisfaction in interaction with situational aspects (Zygar, Hagemeyer, Pusch, & Schönbrodt, 2018b). Thus, there is evidence that partner-related agentic and communal motivation are indeed relevant for the study of romantic relationships at a process level.

In addition to motivational variables, we included relationship satisfaction in our analyses of variability. On the one hand, relationship satisfaction as an indicator of partners’ broad evaluations of their relationship quality is a primary outcome in many studies in couple research (Karney & Bradbury, 1995). Therefore, information on the time scale, levels of its variability, as well as reliability information will be of interest for relationship researchers. On the other hand, relationship satisfaction seems to display some motivational properties as well. In an experience sampling study with 115 couples (six daily assessments over one week), Hofmann, Finkel, and Fitzsimons (2015) found that day-to-day variations in goal progress were positively predicted by variations in relationship satisfaction. Moreover, this effect was mediated by positive affect, perceived partner support, perceived control, and goal focus. Thus, experiences of relationship satisfaction may support the successful implementation of motivational states by fostering a positive self-regulatory mindset.

In our analyses of the time scale and levels of variability regarding the three focal variables agency motivation, communion motivation, and relationship satisfaction, we pursued four research goals: (1) Extend an existing reliability model (Cranford et al., 2006; Shrout & Lane, 2012) with an additional temporal level (moments within a day) and dyadic interdependence (persons nested in couples). (2) Do a variance decomposition that informs on which level (between moments within a day, between days, between persons, between couples) the most variance of relationship motivations and satisfaction is located. (3) Estimate the reliability of relationship motivations and satisfaction on several levels of aggregation (within-person/between-moments, within-person/between-days, between-persons, and between-couples). (4) Evaluate one aspect of the scales’ validity by inspecting scale intercorrelations at the four levels of aggregation.

## Methods

Source code for all statistical models and reproducible analyses are available at the Open Science Framework (https://osf.io/jmeaw/). Raw data for both studies are available as scientific use files (Sample 1: Zygar, Hagemeyer, Pusch, & Schönbrodt, 2018a; Sample 2: Zygar-Hoffmann, Hagemeyer, Pusch, & Schönbrodt, 2020).

### Samples

Data from two intensive experience sampling studies were used. Sample 1 (henceforward, S1) uses a data set from Zygar, Hagemeyer, Pusch, & Schönbrodt, (2018b) which is available as a scientific use file (Zygar, Hagemeyer, Pusch, & Schönbrodt, 2018a). This data set includes ESM data from 130 German-speaking participants (52% women) nested in 68 heterosexual couples. Participants’ mean age was 22.4 years, and the majority (78%) were students. Individuals were on average 2.35 years in a relationship, the majority was not married (97%), and only one participant had children. For a more detailed description of the data set, see Zygar, Hagemeyer, Pusch, and Schönbrodt (2018b).

Sample 2 (S2) includes ESM data from 508 German-speaking participants (50% women) nested in 258 heterosexual couples. Participants were mostly non-students (71%), but held a high school degree (German Abitur) or a higher educational degree (65%). Mean age was 31.4 years and individuals were on average 6.43 years in a relationship. The majority was not married (67%) and had no children (68%).

### Procedure

In both studies, individuals completed an entry questionnaire (programmed with *formr*; Arslan, Walther, & Tata, 2019) on various measures. In the 14 days (S1) or 28 days (S2) that followed, they took part in an experience sampling study, where they answered questions five times a day on their own smartphones, summing up to 9100 scheduled surveys in S1 and 71400 scheduled surveys in S2. The surveys were scheduled semi-randomly across the day, at identical time points for both partners, but during a time-period which couples chose at study registration. Both studies used self-developed ESM apps. For technical reasons, in S1 only individuals with an Android device could participate. In S2 both Android and iOS users could participate. In S1, the first ESM day could be any day of the week. In S2, all participants started their ESM procedure on a Monday (although, due to a continuous enrollment, on several Mondays across a period of eight months).

The surveys were completed in a median time of 3.28 min (S1) and 2.70 min (S2). When notified, individuals had 45 min to complete the survey, which included the same questions at each assessment. An exception was the last survey in the evening in S2. This survey had a different set of items (e.g., did not include the motivation items that are investigated here), and could be completed within five hours, as individuals were instructed to answer it before going to sleep. The average response rate before data exclusions was 84% (S1) and 88% (S2), incentivized by personalized feedback, course credit or money. For more detailed descriptions of the procedures including exclusions we refer the reader to Zygar et al., (2018b) and Zygar-Hoffmann, Pusch, Hagemeyer, and Schönbrodt (2020). Beyond the exclusions documented there, one additional couple was excluded as multiple flags for invalid responding showed up (see https://osf.io/6v2rw/).

### Experience sampling items

**State motivation** At each measurement occasion in S1 and in the first four occasions in S2, three motivational state scales were assessed (see Tables 9 and 10 in the Appendix for all items, instructions and response scales). Communal motivation was assessed with four items at each moment (two Likert scale items and two slider items), for example “How emotionally close would you want to be to your partner at the moment?”. For independence motivation, two items were used, for example “Right now, do you wish: To solitarily pursue your own interests?”. Power motivation was assessed with two (S1) or three items (S2), for example “Right now, do you wish: To influence the feelings or behavior of your partner in any way?”. A fourth scale, referred to as state agency motivation, was computed by summing up independence and power motivation.

**State relationship satisfaction** State relationship satisfaction was assessed with two (S1) or three items (S2) at each moment (see Table 1). Exploratorily, we also constructed a more homogenous two-item scale in S2 by excluding the “annoyance” item, which showed the lowest correlations with the other items (this resulted in a different two-item set than in S1). All reported results concerning S2 refer to the full three-item scale, except the reliability and correlation analyses where results for the two-item scale are additionally reported.

**Table 1:** Experience sampling items for the assessment of relationship satisfaction

Label	Instruction	Scale
Need	Original: **Wie fühlen Sie sich** *jetzt gerade*	Continuous slider from
satisfaction	**in Ihrer Partnerschaft?**	0 = *total frustriert [totally frustrated]*,
(RS-1)	[Englisch: **How are you feeling** *at the moment*]	over
	**in your relationship?**	5 = *neutral [neutral]*, to
		10 = *total erfüllt [totally satisfied]*
Relationship	Original: **Wie geht es Ihnen** *jetzt gerade*	Continuous slider from
mood	**mit Ihrer Beziehung?**	1 (S1) or 0 (S2) = *schlecht [bad]*, over
(RS-3)	[English: **How do you feel about your relationship**	3.5 (S1) or 5 (S2) = *neutral [neutral]*, to
	***at the moment****?*]	7 (S1) or 10 (S2) = *außergewöhnlich*
		*gut [exceptionally good]*
Annoyance	Original: **Wie genervt sind Sie** *jetzt gerade*	Continuous slider from
(RS-4)	**von Ihrem Partner?**	1 (S1) or 0 (S2) = *überhaupt nicht*
	[English: **How annoyed are you about your**	*[not at all]*, to
	**partner** *at the moment?]*	7 (S1) or 10 (S2) = *stark [strongly]*

S1 = Sample 1, S2 = Sample 2. The need satisfaction item (RS-1) was not assessed in S1. The annoyance item (RS-4) was reverse coded for scale calculation. These items can be reused under a CC-BY4.0 license

Several other items were assessed during experience sampling, see the primary documentation of the data sets for a full list of items (see https://osf.io/b8pu6 and https://osf.io/psqx8).

### Statistical procedure

Different models for estimating reliability in intensive longitudinal measures have been proposed (Shrout & Lane, 2012; Cranford et al., 2006; Schoebi, 2008; Nezlek, 2016). Our model is based on generalizability theory (Cronbach, Gleser, Nanda, & Rajaratnam, 1972; Shavelson & Webb, 1991) and extends the Cranford et al., (2006) model with another level of measurement (the order of moments crossed with days) and dyadic interdependence (persons nested in couples). We implemented the model as a random effects intercept-only model to decompose the variance of item responses, allowing to allocate the sources of variances to several temporal levels and multiple other factors. From the same variance decomposition, reliability estimates can be derived based on generalizability theory (Cranford et al., 2006; Shrout and Lane, 2012). Computationally, we estimated variance components using the *lme4* package (Bates, Mächler, Bolker, & Walker, 2015) for linear mixed-effects models in the R environment for statistical computing (Core Team, 2020), where a random intercept variance was estimated for each factor in Eq. 1. We used maximum likelihood (instead of the default restricted ML) because the estimates were more stable (i.e., less dependent on starting values) for our current datasets. The specific function call is in the reproducible scripts on the OSF.

We defined dyad members as nested in couples, and we treated them as indistinguishable (Kenny, Kashy, & Cook, 2006). In our research on motivation in couples, we generally start with the presumption that motivational processes do not significantly differ between men and women (see, for example, Zygar et al.,, 2018b), and try to constrain effects to be equal for both genders. If partners are treated as indistinguishable in the variance decomposition, any systematic between-gender variance is captured by the person factor. From a personality perspective this makes sense to us, as gender differences *are* interindividual differences when looking at persons. Other research foci, however, might prefer to treat partners as distinguishable and to explicitly model a gender factor and its interactions.

**Variance decomposition** Conceptually, level 1 (L1) models the mean of the item responses, which are assessed at each moment (L2), which are crossed with days (L3), which are crossed with persons (L4), which are nested under couples (L5). Following generalizability theory, the full variance decomposition model is formalized as a four-way analysis of variance. For a person *p* nested in couple *c*, responding to item *i* in moment *m* on day *d*, the model for, say, communal motivation *Y*_*cpdmi*_ is

1 $$ \begin{array}{@{}rcl@{}} Y_{cpdmi} &= &\mu + C_{c} + P_{p(c)} + D_{d} + M_{m} + I_{i} \\ &&+(CD)_{cd} + (CM)_{cm} + (CI)_{ci} + (PD)_{p(c)d} \\ &&+(PM)_{p(c)m} + (PI)_{p(c)i} + (DM)_{dm} + (DI)_{di}  \\ &&+ (MI)_{mi} +(CDM)_{cdm} + (CDI)_{cdi} + (CMI)_{cmi} \\ &&+ (PDM)_{p(c)dm} +(PDI)_{p(c)di} + (PMI)_{p(c)mi}  \\ &&+ (DMI)_{dmi}+(CDMI)_{cdmi} \\ &&+e_{cpdmi}. \end{array} $$

Uppercase variables denote the factors *couple (C)*, *person (P)*, *day (D)*, *moment (M)*, and *item (I)*. Subscripts with parentheses denote the nesting structure, for example *P*_*p*(*c*)_ to indicate that persons are nested in couples. In our design, the four-way interaction (*PDMI*)_*p*(*c*)*dmi*_ cannot be distinguished from the error term, because we have no replicate measurements for that interaction. Therefore, the term is subsumed under the error term and does not appear in Eq. 1. Compared to the full five-factorial model, seven terms that include a *couple x person* interaction are missing. As every person is nested under only one specific couple unit, there can be no interaction effect, and consequently such a model would not converge.

The indicator variable for *moment*, *m*, goes from 1 to 5 (S1) or 1 to 4 (S2), which means that *m* = 1, for example, denotes all morning surveys across all persons. The indicator variable for *day*, *d*, goes from 1 to 14 in S1, or 1 to 28 in S2. Hence, *d* = 1 denotes the first study day of all persons. Note that couples started the study on different calendar days in S1. Therefore factor *D* does not capture events which are specific to the calendar day or a specific weekday across participants, but rather systematic variance due to the onset and duration of the study. In S2, couples always started on a Monday, across multiple months. Here, factor *D* can additionally capture systematic weekend effects, as days 7, 14, 21, and 28 are Sundays for each participant. The person indicator *p* runs across couples to reflect the nested data structure (i.e., persons 1 and 2 belong to couple 1, persons 3 and 4 to couple 2, etc.). See Table 2 for an exemplary data structure.

**Table 2:** Exemplary data structure

couple_uid	person_uid	studyday_id	moment_id	item	value
1	1	1	1	1	4
1	1	1	1	2	5
1	1	1	2	1	5
1	1	1	2	2	4
1	1	2	1	1	5
1	1	2	1	2	1
1	1	2	2	1	6
1	1	2	2	2	4
1	2	1	1	1	5
1	2	1	1	2	4
1	2	1	2	1	3
1	2	1	2	2	5
1	2	2	1	1	2
1	2	2	1	2	5
1	2	2	2	1	5
1	2	2	2	2	2
2	3	1	1	1	5
2	3	1	1	2	6
2	3	1	2	1	3
2	3	1	2	2	4
2	3	2	1	1	5
2	3	2	1	2	4
2	3	2	2	1	4
2	3	2	2	2	2
2	4	1	1	1	2
2	4	1	1	2	5
2	4	1	2	1	5
2	4	1	2	2	3
2	4	2	1	1	6
2	4	2	1	2	5
2	4	2	2	1	4
2	4	2	2	2	1
3	5	1	1	1	4
3	5	1	1	2	5
3	5	1	2	1	5
3	5	1	2	2	4
3	5	2	1	1	5
…	…	…	…	…	…

Exemplary reduced data structure for persons in couples, answering two items on two moments on two days (couples could start on a different calendar date and moment 1 could be at different time points for each couple). Indexes for day are repeated within each person, and indexes for moment are repeated within each day of each person (as they are crossed). Indexes for couple and person, in contrast, are unique for each couple and each person in the sample (*uid*= unique id), as persons are nested in couples. The column *value* contains the item responses before standardization

The specific values for the number of items ($i = 1 {\dots } j$), the number of moments nested within each day ($m = 1 {\dots } l$), and the number of days ($d = 1 {\dots } k$) is given in Table 3.

**Table 3:** Design settings

	RS2	RS3	Ind	Pow	A	C
Sample 1						
Number of items *j*	2	–	2	2	4	4
Number of days *k*	14	–	14	14	14	14
Number of moments *l*	5	–	5	5	5	5
Answered surveys	7545	–	7515	7508	7515	7544
Items	RS-3, RS-4	–	I-1, I-2	P-1, P-2	I-1, I-2, P-1, P-2	C-1, C-2, C-3, C-4
Sample 2						
Number of items *j*	2	3	2	3	5	4
Number of days *k*	28	28	28	28	28	28
Number of moments *l*	5	5	4	4	4	4
Answered surveys	60917	60917	47878	47871	47878	47913
Items	RS-1, RS-3	RS-1, RS-3, RS-4	I-1, I-2	P-1, P-2, P-3	I-1, I-2, P-1, P-2, P-3	C-1, C-2, C-3, C-4

*j*, *k*, and *l* are the numbers of scheduled items, days, and moments. Numbers of actually answered slightly differ within study when participants skipped a survey and only partial surveys were recorded. *RS2, RS3*= relationship satisfaction scale, measured with 2, resp. 3, items; *Ind* = independence motivation scale; *Pow* = power motivation scale; *A* = agentic motivation scale (pooled independence and power); *C* = communal motivation scale. RS-1 = need satisfaction item, RS-3 = relationship mood item, RS-4 = annoyance item. For specific item wordings, see Tables 1, 9, and 10

A priori, we did not expect substantial systematic variation for some factors of the design. For example, we did not expect systematic effects for the *day* factor *D* in S1, as persons started on different calendar days. Day 7 of person A presumably has nothing specific in common with day 7 of person B, if these persons are from different couples (except that the same amount of time has passed in the study). In S2, in contrast, all participants started on a Monday. In this case, weekend effects would show up in this factor. Likewise, we did not expect that a certain item has a specific meaning on certain days (*DI* interaction), or on certain moments in general (*MI* interaction), or on certain days for certain persons (*PDI* interaction). Nonetheless, given that we have no empirical evidence for these guesses, we decided to run a factorial model which includes all possible (up to four-way) interactions. This maximal model allows to freely estimate all possible variance components in an explorative way and to see whether certain sources of variances indeed are (close to) zero.

Several conceptually meaningful units emerge in the model as interactions between factors. For example, the three-way interaction *person x day x moment*, *PDM*, refers to specific surveys of specific persons on specific days (e.g., *p* = 5, *d* = 2, *m* = 1, refers to the morning survey of person 5 on her second day). The variance of this component quantifies the variability between these specific surveys across all moments of all participants (averaging across all items). The meaning of the other components together with an explanation of their respective variance components can be found in Table 4.

**Table 4:** Variance decomposition of item responses: meaning of terms

Source	Explanation	Example/Comment
Theoretically relevant terms		
couple (C)	Variance between couples	
person (P)	Variance between persons	
day (D)	Variance between days 1 to 14/28	Time trends across the study, or effects
	(pooled acrossed all persons)	of weekend vs. weekday.
moment (M)	Variance between time points 1 to 5	Systematic effects of morning vs.
	(moments are pooled within and across all persons)	evening
couple:day (CD)	Do specific days have different meanings	Shared daily characteristics (e.g.
	for each couple? (days 1 to 14/28)	being together on a family gathering)
person:day (PD)	Variance between days (each day of each	
	person is a unique day)	
couple:day:moment (CDM)	Event-level variance between couples	Shared momentary environment
person:day:moment (PDM)	Variance between moments (each	
	moment of each person is unique)	
Nuisance terms		
item (I)	Do the mean level of items differ?	Items are z-standardized, therefore we
		expect only small values
couple:item (CI)	Do couples have a stable, differential	Couples agree on a common
	understanding of items?	understanding of specific items
couple:moment (CM)	Systematic effects of moment for some	Systematic effects of morning vs.
	couples	evening for some couples
moment:item (MI)	Do specific items have a specific	All persons change the interpretation of
	meaning on specific time points of the day,	some items in the evening.
	pooled across all days of all persons?	
person:item (PI)	Do persons have a stable, differential	Differential item functioning for men
	understanding of items?	and women, or for specific persons
person:moment (PM)	Variance between time points of a day	Systematic effects of morning vs.
	(pooled within each person)	evening for some persons
day:item (DI)	Do specific items have a specific meaning	All persons change the interpretation of
	on specific days, across all persons?	some items on fridays (assumed that all
		participant started on a Monday).
day:moment (DM)	Do certain events (e.g., moment 4 on	All persons report higher values on all
	day 9) have a special meaning across all persons?	items on the first moment of the first ESM day.
couple:moment:item (CMI)	Do couples have a stable, differential	Couples differ in their shared
	understanding of items at specific time points	understanding of items in the morning vs.
	across all days?	in the evening.
couple:day:item (CDI)	Do couples have a stable, differential	Some couples change the
	understanding of items at specific days?	interpretation of some items at specific days
person:moment:item (PMI)	Do person have a differential	Some persons change the interpretation
	understanding of items on specific time points	of some items in the evening
	(1 to 5) across all days?	
person:day:item (PDI)	Do persons have a differential	Some persons change the interpretations
	understanding of items at specific days	of items on specific days
	(1 to 14/28)?	
day:moment:item (DMI)	Do specific items have a specific	All persons change the interpretation
	meaning on specific moments of specific	of an item on the evening of ESM day 6.
	days (across all persons)?	
couple:day:moment:item (CDMI)	Do couples have a stable, differential	Different understanding of items after a
	understanding of items at specific time	conflict between the partners
	points of specific days?	
Error (e)	Residual error variance	

For estimating the model, several assumptions have to be made (Shrout & Lane, 2012): (a) Errors and true scores are independent, which also implies that no autoregressive effects are present, (b) the variances are fixed (i.e., the same for all units), (c) items have the same weight of the latent factor. There are good reasons why these assumptions do not reflect realistic properties of psychological data, and the consequences of violating them is discussed exemplarily for the current data in the limitation section.

**Data preprocessing** The items of our communion motivation scale were assessed on different response scales. The GT model covers differing mean levels of items with the item factor *I*. However, different scales can also pose (additional) problems for the assumption of equal item loadings and the assumption of fixed variances. In practice, items with different response options are typically averaged to a scale score by first standardizing them.^1^ As we wanted to match our reliability analysis to the actually computed scale scores, we *z*-standardized all items across all measurement points of both genders. (The reliability estimates from unstandardized variables were virtually identical). Furthermore, we recoded one reversed item for relationship satisfaction (RS-4, see Table 1).

**Reliability estimation**

Reliability estimation in the GT framework generally uses the formula

2 $$  R_{X} = \frac{ {\sigma^{2}_{T}} }{ {\sigma^{2}_{T}} + {\sigma^{2}_{e}}} $$

where ${\sigma ^{2}_{T}}$ is the variance of the true scores and ${\sigma ^{2}_{e}}$ is the variance of the random measurement error, which is assumed to be constant across units and replications (Shrout & Lane, 2012).

Based on this general reliability approach, Cranford et al., (2006) and Shrout and Lane (2012) derived formulas that compute reliability on several levels in experience sampling designs. Here, we extend these formulas with an additional temporal level (moments crossed with days) and dyadic interdependence.

For all following reliability formula, we assume that days, *D*, are random (and not fixed), because participants started on different days across a period of several months, and the study period is not contingent on some common event. Moments, *M*, in contrast, were treated as fixed, as the moments each day (from morning to evening) were assumed to be comparable for each person.^2^ Finally, the item factor, *I*, is treated as fixed (cf. Shrout & Lane, 2012), as no generalization beyond this specific item set is aimed for. Consequently, ${\sigma ^{2}_{M}}$, ${\sigma ^{2}_{I}}$, and $\sigma ^{2}_{MI}$ play no role in the following reliability formulas.

Depending on the focal level for which reliability should be assessed, different terms contribute to the numerator (the true score variance) and the denominator (the observed variance). Generally, terms located on a higher level that do not vary within the focal level do not contribute to reliability estimation. For example, if we are interested in the measurement of purely within-person changes, the variance of the term *PI* (i.e., *person x item*), $\sigma ^{2}_{PI}$, neither contributes to systematic variances nor to the error term, as mean level biases in item understanding between persons are irrelevant for relative within-person assessments: Within each person, this is a constant mean level shift that does not contribute to variations within that person. Likewise, systematic variance between days of a person, $\sigma ^{2}_{PD}$, is an irrelevant source of variance if moment-to-moment change within a day is assessed, and between-couple variance, ${\sigma ^{2}_{C}}$, does not contribute to between-person reliability estimation or any other lower level.

For the *numerator*, one starts with a focal level for which reliability should be assessed, for example “between persons”. The numerator contains all sources of systematic variance for that level. In our example, this primarily is the person factor *P*, which contains all between-person variance. However, depending on which factors are a priori defined as fixed, some additional interaction terms also contribute to systematic between-person variance. Typically, just as it is in our case, the item factor *I* is considered fixed. Consequently, the *person x item interaction* *PI* contains idiosyncratic response patterns. If person A has on average higher scores on item 1 than would be expected by the main effects of the person mean and the item mean, this contributes to systematic between-person differences. As we assume moments *M* to be fixed, the *person x moment* interaction *PM* must be considered, too: If person B is not a morning person and always responds lower on all items in the morning survey, this variance component also contains systematic between-person variance. The same logic applies to the *PMI* interaction. Hence, the numerator contains the focal random factor, and all interactions of fixed factors with this random factor.

The *denominator* is the sum of systematic plus random (error) variance. Hence, along with all terms of the numerator, it contains all other random terms (including interactions with at least one random term) which are not on a higher level than the focal level. In the GT reliability computation, variance components are divided by the number of replications that are averaged when aggregating the scale scores, in order to account for the increased precision when more measurements are available (see the explanations after the formula in the next paragraph).

Based on these assumptions, *between-couple reliability* (averaging all measurements of both persons of a couple across the entire study), *R*_*BC*_, can be defined as:

3 $$ \begin{array}{@{}rcl@{}} R_{BC} = \frac{ {\sigma^{2}_{C}} + [\sigma^{2}_{CI} / j] + [\sigma^{2}_{CM} / l] + [\sigma^{2}_{CMI} / (l*j)] }{ \begin{array}{l} {\sigma^{2}_{C}} + [\sigma^{2}_{CI} / j] + [\sigma^{2}_{CM} / l] + [\sigma^{2}_{CMI} / (l*j)] + [\sigma^{2}_{CD} / k] \\ + [\sigma^{2}_{CDM} / (k*l)] + [\sigma^{2}_{CDI} / (k*j)] + [\sigma^{2}_{CDMI} / (k*l*j)] \\  + [{\sigma^{2}_{P}} / 2] + [{\sigma^{2}_{D}} / k]+ [\sigma^{2}_{PD} / (2*k)] + [\sigma^{2}_{PM} / (2*l)] \\ + [\sigma^{2}_{PI} / (2*j)] + [\sigma^{2}_{DM} / (k*l)] + [\sigma^{2}_{DI} / (k*j)]\\ + [\sigma^{2}_{PDM} / (2*k*l)] + [\sigma^{2}_{PDI} / (2*k*j)] + [\sigma^{2}_{PMI} / (2*l*j)]\\ + [\sigma^{2}_{DMI} / (k*l*j)] + [{\sigma^{2}_{e}} / (2*k*l*j)] \end{array} }\\ \end{array} $$

Constant *j* is the number of items, *k* is the number of days, *l* is the number of moments within each day (see Table 3 for the specific values). Each variance component is divided by the number of replications. For example, the *couple x item* variance is divided by *j*, as for each couple *j* estimates, for each level of the item factor, are considered. The residual error term ${\sigma ^{2}_{e}}$ in *R*_*BC*_ is divided by 2 ∗ *k* ∗ *l* ∗ *j* to take into account the increase in precision that results from averaging *j* items, assessed at *l* moments at each of the *k* days for both (i.e., two) persons in each couple. Finally, note that the variance components for I, M, and MI do not appear in the denominator as we assumed them to be fixed.

For computing *between-person reliability* (averaging all measurements of a person across the entire study), *R*_*BP*_, we extend Equation (8) from Shrout and Lane (2012) by the new temporal level of moments, and all necessary interactions of the new moment factor with other factors:^3^

4 $$ \begin{array}{@{}rcl@{}} R_{BP} = \frac{ {\sigma^{2}_{P}} + [\sigma^{2}_{PI} / j] + [\sigma^{2}_{PM} / l] + [\sigma^{2}_{PMI} / (l*j)] }  {\begin{array}{l}\sigma^{2}_{P} + [\sigma^{2}_{PI} / j] + [\sigma^{2}_{PM} / l] + [\sigma^{2}_{PMI} / (l*j)]+ [{\sigma^{2}_{D}} / k]\\ + [\sigma^{2}_{PD} / k] + [\sigma^{2}_{DM} / (k*l)] + [\sigma^{2}_{DI} / (k*j)]+ [\sigma^{2}_{PDM} / (k*l)] \\ + [\sigma^{2}_{PDI} / (k*j)] { + [\sigma^{2}_{DMI} / (k*l*j)] + [{\sigma^{2}_{e}} / (k*l*j)] } \end{array}}\\ \end{array} $$

We computed *within-person change reliability from day to day*, *R*_*WPD*_ (averaging over *l* moments within a day), as:

5 $$ \begin{array}{@{}rcl@{}} R_{WPD} = \frac{ \sigma^{2}_{PD} + [\sigma^{2}_{PDI} / j] + [\sigma^{2}_{PDM} / l] }{ \begin{array}{l}\sigma^{2}_{PD} + [\sigma^{2}_{PDI} / j] + [\sigma^{2}_{PDM} / l] + {\sigma^{2}_{D}} + [\sigma^{2}_{DM} / l] \\ {+ [\sigma^{2}_{DI} / j] + [\sigma^{2}_{DMI} / (l*j)] + [{\sigma^{2}_{e}} / (l*j)]} \end{array}}\\ \end{array} $$

On the lowest temporal level *within-person change reliability from moment to moment*, *R*_*WPM*_, is computed as (cf. Shrout & Lane, 2012, Eq. 9):

6 $$  R_{WPM} = \frac{ \sigma^{2}_{PDM} }{ \sigma^{2}_{PDM} + [{\sigma^{2}_{e}} / j] } $$

The number of days within person, *k*, and the number of moments within day, *l*, is not constant if participants do not answer every single ESM survey. Therefore, for the actual computation of all reliabilities we inserted the average number of answered moments (i.e., response rate x maximum possible observations) and the average number of days into the formulas (see also Scott et al.,, 2018, footnote 5, and Shrout & Lane, 2012).

**Application of reliability formulas to related data structures**

The provided formulas can be adapted to related data structures. For measurement designs without a dyadic structure on the highest level, reliability formulas *R*_*BP*_, *R*_*WPD*_, and *R*_*WPM*_ are identical. In this case, the variance decomposition in Eq. 1 simply omits all terms including the factor *C*.

For measurement designs with a dyadic structure but with only a single daily measurement, the variance decomposition in Eq. 1 omits all terms including the factor *M* and the term *PDI*, as the latter cannot be distinguished from the error term, because no replicate measurements are present for that interaction. The between-person reliability formula simplifies to:

7 $$  R_{BP\_nondyadic} = \frac{ {\sigma^{2}_{P}} + [\sigma^{2}_{PI} / j] }{\begin{array}{l}{\sigma^{2}_{P}} + [\sigma^{2}_{PI} / j] + [{\sigma^{2}_{D}} / k] + [\sigma^{2}_{PD} / k] \\ + [\sigma^{2}_{DI} / (k*j)] + [{\sigma^{2}_{e}} / (k*j)] \end{array}} $$

Note that, in contrast to Eq. 8 in Shrout and Lane (2012), we added $\sigma ^{2}_{DI} / (k*j)$ to the denominator, as time is considered to be random.

The within-person change reliability from day to day simplifies to:

8 $$  R_{WPD\_nondyadic} = \frac{ \sigma^{2}_{PD} }{ \sigma^{2}_{PD} + {\sigma^{2}_{D}} + [\sigma^{2}_{DI} / j] + [{\sigma^{2}_{e}} / j] } $$

Note that, in contrast to Eq. 9 in Shrout and Lane (2012) and Eq. 5 in Cranford et al., (2006), we added ${\sigma ^{2}_{D}}$ and $\sigma ^{2}_{DI} / j$ to the denominator, as time is considered to be random.

**Scale intercorrelation at four levels of aggregation** We computed correlation matrices of all scales on the four conceptual levels. Non-independence of data due to the hierarchical structure was handled by controlling for mean differences of all higher level units: (a) Scale scores on the *between-couple level* were computed by averaging all item responses of a scale across all measurements of both persons in a couple. (b) Scale scores on the *between-person level* were computed by subtracting the couple means from all answers and averaging the residuals across all measurements of each person. (c) Scale scores on the *within-person/between-days level* were computed by sequentially subtracting the couple and the person means and averaging the residuals across all measurements of each day of a person. (d) Scale scores on the *within-person/between-moments level* were computed by sequentially subtracting the couple, person, and day means and averaging the residuals within each moment of a person.

Centering the item responses to the mean of all higher units removes potential confounding effects. For example, for the between-days analysis, all potentially confounding between-couple and between-person effects are controlled for by removing the respective means from the item responses. After this preprocessing, correlations were computed across the full sample.

## Results

### Variance decomposition

Table 5 reports the absolute variance estimates and Table 6 reports a relative variance partitioning of the systematic (non-error) variances. For a better overview, we categorized sources of variance into “theoretically relevant terms” (i.e., of substantive interest) and “nuisance terms”, although some of the terms that we consider nuisance terms here might be centrally relevant for other research questions (e.g., for methodological and psychometric questions).

**Table 5:** Variance decomposition of item responses: absolute variances

	Sample 1, absolute					Sample 2, absolute				
Variance component *σ*^2^	RS*	Ind	Pow*	A*	C	RS*	Ind	Pow*	A*	C
Theoretically relevant terms										
couple (C)	.10	.04	.07	.05	.10	.20	.08	.06	.06	.16
person (P)	.08	.19	.11	.10	.18	.10	.16	.19	.12	.11
day (D)										
moment (M)		.01			.01					
couple:day (CD)	.09	.01		.01	.01	.08	.02	.01	.01	.02
person:day (PD)	.01	.11	.05	.02	.06	.06	.08	.07	.04	.07
couple:day:moment (CDM)	.11	.03	.01		.04	.09	.02			.03
person:day:moment (PDM)	.08	.17	.11	.04	.15	.09	.13	.13	.05	.12
Nuisance terms										
item (I)										
couple:item (CI)	.04	.02	.03	.03	.03	.04	.02	.02	.03	.04
couple:moment (CM)		.01								
moment:item (MI)				.01						
person:item (PI)	.12	.04	.20	.17	.08	.08	.08	.12	.16	.13
person:moment (PM)		.01			.01		.01			
day:item (DI)										
day:moment (DM)										
couple:moment:item (CMI)										
couple:day:item (CDI)	.02					.01			.01	.01
person:moment:item (PMI)				.01	.01					
person:day:item (PDI)	.05	.03	.08	.11	.05	.04	.05	.06	.10	.06
day:moment:item (DMI)										
couple:day:moment:item (CDMI)	.02			.02	.01	.01			.01	.01
Error (e)	.28	.34	.33	.43	.27	.19	.33	.32	.40	.24

*RS* = relationship satisfaction scale, *Ind* = independence motivation scale, *Pow* = power motivation scale, *A*= agentic motivation scale (pooled independence and power), *C* = communal motivation scale. Empty cells have values <.005. Scales marked with an asterisk do not contain the same items in S1 and S2

**Table 6:** Variance decomposition of item responses: relative systematic variances

	Sample 1, relative in %					Sample 2, relative in %				
Source	RS*	Ind	Pow*	A*	C	RS*	Ind	Pow*	A*	C
Theoretically relevant terms										
couple (C)	13	6	10	8	13	25	12	9	9	21
person (P)	11	28	17	17	24	12	23	28	20	14
day (D)				1						
moment (M)		2			1					
couple:day (CD)	12	1		1	2	10	3	2	1	3
person:day (PD)	1	16	8	3	9	7	13	10	6	9
couple:day:moment (CDM)	16	5	1		5	11	3		1	3
person:day:moment (PDM)	11	25	16	7	21	11	19	19	8	15
Nuisance terms										
item (I)										
couple:item (CI)	5	3	4	6	4	5	3	3	6	5
couple:moment (CM)		1								
moment:item (MI)				1						
person:item (PI)	17	6	29	30	10	10	13	18	27	17
person:moment (PM)		1	1		2		1			
day:item (DI)										
day:moment (DM)										
couple:moment:item (CMI)										
couple:day:item (CDI)	3					2			2	1
person:moment:item (PMI)				1	1				1	
person:day:item (PDI)	7	4	12	20	7	5	8	9	16	8
day:moment:item (DMI)										
couple:day:moment:item (CDMI)	3			4	1	2			1	1

*RS* = relationship satisfaction scale, *Ind* = independence motivation scale, *Pow* = power motivation scale, *A*= agentic motivation scale (pooled independence and power), *C* = communal motivation scale. Empty cells have values <.05%. Scales marked with an asterisk do not contain the same items in S1 and S2

As a general pattern, four focal sources of variances had the largest share across scales and studies: persons (*P*; around 19% of systematic variance), specific moments of persons (*PDM*; around 15%), couple (*C*; around 13%), and specific days of persons (*PD*; around 8%). Beyond these general trends, however, specific variance components are more pronounced in some scales than others. For example, the largest share of couple-level variance is mostly present in relationship satisfaction and communal motivation. Furthermore, relationship satisfaction additionally has a unique large *couple x day* component (*CD*; around 11%) and *couple x day x moment* component (*CDM*; around 14%), which indicates that some days and some specific moments are more satisfying for some couples than other days or moments.

Concerning nuisance terms, two sources of variances had substantial contributions across scales and studies: After controlling for between-person variance, participants still had systematically different mean levels between item responses in general (*PI*; around 18% of variance), and on specific days (*PDI*; around 10%).

### Reliability estimation

Table 7 reports reliability estimates for both studies on all levels. On couple level, reliabilities range from .32 to .76, on person level from .93 to .98, on day level from .61 to .88, and on moment level from .28 to .72.^4^

**Table 7:** Reliability estimates

	Sample 1				Sample 2			
Scale	*R*_*BC*_	*R*_*BP*_	*R*_*WPD*_	*R*_*WPM*_	*R*_*BC*_	*R*_*BP*_	*R*_*WPD*_	*R*_*WPM*_
RS2*	.58	.95	.61	.36	.74	.97	.88	.65
RS3					.76	.97	.86	.58
Ind	.32	.93	.79	.50	.46	.96	.74	.44
Pow*	.43	.95	.73	.40	.37	.98	.79	.54
A*	.44	.96	.66	.28	.44	.98	.73	.38
C2	.42	.95	.88	.72	.58	.97	.86	.70
C3	.47	.96	.85	.65	.66	.97	.85	.63
C4	.50	.95	.87	.70	.70	.97	.86	.67

*R*_*BC*_ = between-couples reliability, *R*_*BP*_ = between-persons reliability, *R*_*WPD*_ = within-person/between-days reliability, *R*_*WPM*_ = within-person/between-moments reliability. *RS2, RS3* = relationship satisfaction scale, measured with 2, resp. 3, items, *Ind* = independence motivation scale, *Pow* = power motivation scale, *A* = agentic motivation scale (pooled independence and power), *C* = communal motivation scale. Scales marked with an asterisk do not contain the same items in S1 and S2. RS2 = relationship mood + annoyance items in S1 and relationship mood + need satisfaction items in S2 (see Table 1)

### Scale correlations on four levels of aggregation

The raw bivariate correlations are not corrected for unreliability of the scales, which has to be kept in mind when comparing the absolute sizes between the three levels. As reliability is lowest on the between-moment level, also lower correlations are to be expected. Table 8 reports the correlations on each level of aggregation.

**Table 8:** Correlations on four levels of aggregation

	RS2	RS3	Ind	Pow	A	C
Between-couple correlations						
RS2			−.47	−.22	−.41	.40
RS3	.97					
Ind	−.16	−.20		.42	.84	−.18
Pow	.02	−.02	.46		.85	.45
A	−.06	−.10	.79	.91		.16
C	.60	.58	−.16	.40	.20	
Between-person correlations						
RS2			−.22	.05	−.11	.40
RS3	.94					
Ind	−.12	−.21		.29	.80	−.15
Pow	−.18	−.27	.35		.81	.58
A	−.19	−.30	.72	.90		.27
C	.30	.27	−.15	.32	.17	
Within-person, between-day correlations						
RS2			−.15	.05	−.08	.37
RS3	.94					
Ind	−.19	−.20		−.05	.74	−.38
Pow	.03	.01	.09		.63	.50
A	−.09	−.11	.65	.82		.04
C	.41	.42	−.30	.40	.13	
Within-person, between-moment correlations						
RS2			−.13	.00	−.10	.28
RS3	.90					
Ind	−.13	−.15		−.06	.75	−.36
Pow	.02	.00	.00		.62	.41
A	−.07	−.09	.61	.79		−.01
C	.33	.33	−.30	.34	.09	

Upper triangle in each matrix shows S1, lower triangle shows S2. *RS2, RS3* = relationship satisfaction scale, measured with 2, resp. 3, items, *Ind* = independence motivation scale, *Pow* = power motivation scale, *A* = agentic motivation scale (pooled independence and power), *C* = communal motivation scale

Generally, the matrices show largely similar patterns across aggregation levels. In particular, all differences between the day level correlations and the moment level correlations are less than .09, with an average absolute difference of .03. The correlations on person level, however, show some stronger differences to the day and moment level correlations. Specifically, the correlation between power and independence motivation is around .32 on the person level, but close to zero on the day and moment level. Furthermore, the negative correlation between independence motivation and communal motivation is stronger on the day and moment level (*r* between −.30 and −.38) compared to the person level (*r* = −.15).

## Discussion

We presented a model for estimating the reliability of experience sampling measures which are assessed at multiple moments per day, across several days, for persons within dyads. This design allows researchers to estimate a variance decomposition and reliability on four levels of aggregation, (a) between-couples, (b) between-persons, (c) within-person/between-days, and (d) within-person/between-moments. The model was applied to estimate variance components and reliabilities of five scales that are central to the study of motivational dynamics and relationship satisfaction in couples: State relationship satisfaction, communal motivation, and agency motivation, which has been assessed with two subscales, independence motivation and power motivation. Two intensive longitudinal studies provided data on more than 7508 unique surveys in Sample 1 and more than 47764 unique surveys in Sample 2.

### Variance decomposition and reliability estimation

One research question for this study was about on which temporal level (between moments within a day, between days, between persons, between couples) most variance of relationship motivations and satisfaction is located. This also allows the investigation of the time scale of variability of motivational processes and relationship satisfaction. Four theoretically relevant sources of variance had the largest share across scales and studies: persons, specific moments of persons, couples, and specific days of persons. That means, some persons and some couples are to some extent generally closer, more satisfied, or have more agentic motivation than other persons or couples. Furthermore, the investigated scales varied both from day to day and from moment to moment. The within-day variance, from moment to moment, was around twice as large as the between-day variance, and nearly as large as the between-person variance. Hence, the pattern of results shows (a) the existence of systematic inter-individual differences in self-reported motivational states and relationship satisfaction, (b) systematic inter-couple differences, that indicate some dyadic similarity in couples, and (c) that these scale values show more short-time variability within a day than variability between days.

Concerning nuisance terms, two sources of variance had substantial contributions across scales (in particular agency motivation) and studies. First, after controlling for between-person differences, participants still systematically demonstrated person-specific mean levels of item responses. This can be due to differential item functioning, which indicates that an item might be measuring different latent constructs for members of different subgroups. Follow-up analyses with explanatory variables, such as gender, marital status, or relationship duration, might reveal which specific subgroups have a differing understanding of items. Second, persons had a differential item understanding on specific days. This can happen, for example, if items are interpreted differently at weekends (vs. workdays) by some persons. From a psychometric point of view, these sources of variance should be as small as possible for a general-purpose questionnaire.

When item responses were aggregated on person level, all scales showed near perfect reliability >.93 (S1) and >.97 (S2). Aggregated on day level (across four or five moments per day), reliability of the more homogeneous scales fell between .73 and .88. The two items for state relationship satisfaction in S1 were quite inhomogenous, resulting in a lower reliability of .61. Furthermore, combining independence and power motivation into a higher-order agency scale decreased reliability to .66 in S1.

On the lowest level of aggregation, at each moment, this trend was even stronger. Homogeneous scales showed (relatively) better reliabilities ranging from .40 to .70. The moment-level reliabilities of the combined agency scale (.28 in S1, .38 in S2) and the two heterogeneous relationship satisfaction items in S1 (.36) were unsatisfactory. Hence, concerning reliability, the two-item relationship satisfaction scale from S2 (with items RS-1 and RS-3) seems preferable to the two-item scale from S1 (with items RS-3 and RS-4). Although the full three-item scale in S2 does not improve reliability compared to the two-item scale, it covers a broader content range and might have better validity. It might thus be preferred, depending on the research question (see Zygar-Hoffmann and Schönbrodt, 2020, for validity considerations associated with this item).

### Validity: Scale intercorrelations

The scale intercorrelations on the different temporal levels revealed some relevant insights into the underlying constructs. Generally, the correlation matrices were rather similar on all levels and did not show strong indicators of a Simpson’s paradox, where associations between variables are very different between aggregation levels or even flip their sign. However, there were two notable exceptions where the person level correlations differed from the day and moment level correlations.

First, the independence and power motivation scales showed a positive correlation around .32 on the between-person level. Persons who generally had more independence motivation also generally had more power motivation, which can be interpreted that these scales are two facets of the overarching agency motive factor, which represents “a superordinate need to feel as a capable, self-reliant individual” (Hagemeyer & Neyer, 2012, p. 3). Within person, however, they were independent with correlations close to zero: On moments or days where persons experienced a strong motivation for independence, they did not necessarily experience a concurrent motivation for power. A theoretically consistent interpretation would be that independence and power are different implementation styles of enacting agency in relationships. Although they do not go together at each moment in time, both are different (and to some extent exchangeable) ways to express a superordinate need for agency.

This correlation structure of the agency subscales has implications both for assessment and theory building. Zero correlations on a momentary level lead to low reliabilities of the combined agency scale. Consequently, unless one is explicitly treating agentic motivation as a formative construct on the day or moment level, we generally recommend not to use that combined scale, but rather to treat both subscales as separate on the day or moment level. On the between-person level, in contrast, the subscales showed a substantial positive correlation, which was also reflected in higher reliabilities of the combined agency scale.

Dissociations of motivational processes and domains at different conceptual levels should also get more attention in theory building. Within-person processes do not necessarily reflect between-person structures, and vice versa (Molenaar, 2008). Consequentely, theory building in motivation ideally covers both levels, and researchers should be careful when inferences and implications are transferred from one level (e.g., within-person experimental manipulations in the lab) to the other level (e.g., between-person structures of motivational domains). This call is in line with previous research that demonstrated differences in between-person and within-person structures of the Big Five personality traits (e.g., Borkenau & Ostendorf, 1998; Grice, Jackson, & McDaniel, 2006) or positive and negative affect (e.g., Brose, Voelkle, Lövdén, Lindenberger, & Schmiedek, 2015).

Second, independence and communal motivation were, to some extent, mutually exclusive on the daily and momentary level (with an *r* around −.34), but not so much on the between-person level (*r* = −.15). On a behavioral level this makes immediate sense, as it is difficult to be (emotionally) close to the partner, and at the same time to independently follow your own interest. On the motivational level, in contrast, such an ambiguity is imaginable, where persons simultaneously want to be close and distant from the partner. Empirically, however, the negative correlation shows that such ambiguous motivational states were rather rare. On the person level, in contrast, the correlation is only slightly negative, indicating that a person’s general level of communal motivation was largely independent of the general level of independence motivation.

When the agency and the communion motive have been assessed as stable dispositions, they typically have shown negative correlations around −.40, both on an explicit level, assessed with self-report questionnaires (Hagemeyer et al., 2013), and on an implicit level, assessed with indirect methods (Hagemeyer & Neyer, 2012). In contrast to these previous results, we found slightly positive correlations of agentic and communal motivation on person level between .17 and .27, and correlations in the range of −.01 to .13 on the moment or day level. This deviation from previous results can partly be explained by the specific conceptualization of the combined agency scale in the current ESM studies. Inspecting the two agency subscales reveals that the independence subscale showed the expected negative correlation to communal motivation on day and moment level, and a weak negative correlation on person level. The explicit agency (dispositional) motive in the studies cited above has been assessed with the ABC scales (Hagemeyer et al., 2013), which focus on the agentic aspect of “forming separations” (Bakan, 1966). Hence, items such as “I like to be completely alone” from the ABC scales are most closely related to the independence motivation items in the current study, which did show the expected negative correlation (albeit, with a smaller effect size).

The positive correlation between power motivation and communal motivation on all levels of aggregation might be due to two different factors. First, some of our ESM power items were inspired by prosocial aspects of the power motive as described in Winter (1994) and Hagemeyer and Neyer (2012), where power motivation includes supportive behaviors/motivations within the relationship as well as a positive influence on the partner. Therefore, our ESM power items focus on prosocial aspects of power and do not address aspects that are usually valued negatively, such as dominance in the relationship. Thus, the power and the communion scale share a common positive connotation. Second, in contrast to independence, the power aspect of agency often requires contact to the partner. Thus, the power and communion items share a common mode of implementation, namely seeking proximity to the partner. One way to further disentangle different facets of agency would be to separately investigate coercive or aggressive dominance as another facet of agency (Suessenbach, Loughnan, Schönbrodt, & Moore, 2019). Dominance motivated instrumental behavior in that sense also requires proximity to the partner, but does not share the same positive connotation as our operationalization of power motivation does.

### Implications for Future Research

The results have some direct implications for the design and the statistical analysis of studies using these scales. First, a considerable amount of variance was located on the between-couple level. Hence, the dyadic structure should not be ignored in statistical analyses. Second, all scales showed more variance between moments (within a day) than between days. Hence, a daily diary, which has only a single measurement per day, probably misses large parts of the fluctuations in these constructs. Third, the analyses revealed an unexpected large amount of differential item functioning between persons, but also between days within persons. This underscores the importance of proper psychometric analyses and intensive pilot testing of the ESM item wordings and how participants understand them. In the current two studies, we did multiple pilot studies where we refined items and asked participants in S1 in a post-ESM-questionnaire how they interpreted the items, using open ended questions. Additionally, in both studies before starting the ESM part, all participants received instructions (written in S1, video-recorded in S2) on how to interpret the items, and could look up the instructions for each item during the study. Despite these efforts, not all persons had the same understanding of items, and we suppose that this source of variance might be even larger in studies that do not have the same amount of pretesting.

Fourth, change reliability on the moment-to-moment level was mostly not satisfactory. When such unreliable scale scores are used as predictors or outcomes in follow-up statistical models, two aspects influence statistical power, working in opposite directions: As reliability is lowest on the most fine-grained moment level, statistical power is lowered. At the same time, this level also has the largest number of measurement points, which in turn increases the statistical power to detect existing effects. For example, despite the low reliability of .36 in the two-item relationship satisfaction scale used in S1, Zygar et al., (2018b) found reliable evidence for hypothesized effects on this outcome variable (see robustness check, footnote 10).

When designing an ESM study, specifically the frequency, timing, and length of measurements, several factors must be considered. The expected rate of change of a construct determines the frequency of sampling, and reliability and burden of participants must be balanced (for further aspects regarding the sampling plans of state relationship satisfaction, see Zygar-Hoffmann and Schönbrodt, 2020). For planning a study, power analyses are needed to investigate the relative impact of these determinants on statistical power.

### Limitations

Several limitations follow from the assumptions that have to be made for computing the variance components (Shrout & Lane, 2012). Most importantly, the components of Eq. 1 are assumed to be independent, which is most likely violated in multiple ways. Although the random intercept for *couple* accounts for some of the dyadic interdependence, it does not model covariances between dyad members. This ignorance of dyadic covariances is acceptable if covariances are positive, as was the case in our data sets. In this case, variances are shifted towards a higher level (e.g., between-person variance gets reallocated to the couple level if persons within a couple are more alike to each other), which makes sense. However, if dyadic covariances are negative, this can lead to estimation problems and/or biased variance estimates. Another likely violation of the independence assumption is that consecutive time points in an ESM presumably have some autoregressive effect, which is ignored in the GT model. Finally, the model assumes equal item loadings. Simulations by Lane and Shrout (2010) showed that the GT method underestimates the reliability to the extent that the assumption of equal item loadings is violated. One way to get closer to equal items loadings can be the standardization of items before calculating the scale, in particular when items do not have the same response scale. This, however, is not always desirable in terms of interpretation.

Bearing these limitations in mind, we think that this model is an acceptable approximation for our current research question. We note, however, that this does not necessarily generalize to other data sets, in particular when negative dyadic covariances are present.

Further, the analysis relates only to our specific operationalization of motivation and relationship satisfaction. The statelikeness of a phenomenon is also a feature of the specific item wording, and a different phrasing might shift the variance components more towards the person or couple level. Furthermore, we only used two to four items per scale. This gave only few possibilities to do item selection. Scale development for ESM studies can benefit from a larger item pool in a pilot study that allows to choose an item set that balances homogeneity and content width. Joint efforts to collect ESM items and curate the documentation of their psychometric quality are another important step that helps to achieve reliable and valid ESM scales (see e.g., http://www.esmitemrepository.com; Kirtley et al.,, 2020).

### Conclusions

Creating items and scales for ESM has some special challenges. Many ESM studies use ad-hoc scales with very few items, and proper psychometric analyses are rarely seen. Here we extend the psychometric toolbox by proposing a variance decomposition and reliability model for data sets where constructs are assessed with multiple items at multiple moments each day in couples. Applying this model to four motivation scales and different scales for state relationship satisfaction showed substantial variability on state level, different reliabilities depending on the level of aggregation, and theoretically interesting patterns of scale intercorrelations. The model can also easily applied to data set where persons are not nested in couples (by removing all terms related to the couple), and we encourage researchers to use the provided R-Scripts on the OSF to calculate reliability analyses of their ESM scales for individual as well as dyadic study designs.

## Open Practices Statement

Due to the dyadic nature of the data set, we cannot make the data fully openly available. The data and materials for Sample 1 (10.5160/psychdata.zrce16dy99) and Sample 2 (10.5160/psychdata.zrce18mo99) are published as scientific use files, which restricts access to scientific users. The reliability analyses presented here were not preregistered. Reproducible scripts for all data analyses reported in this paper are available at the Open Science Framework (https://osf.io/jmeaw/).

## Figures and Tables

**Table 9:**
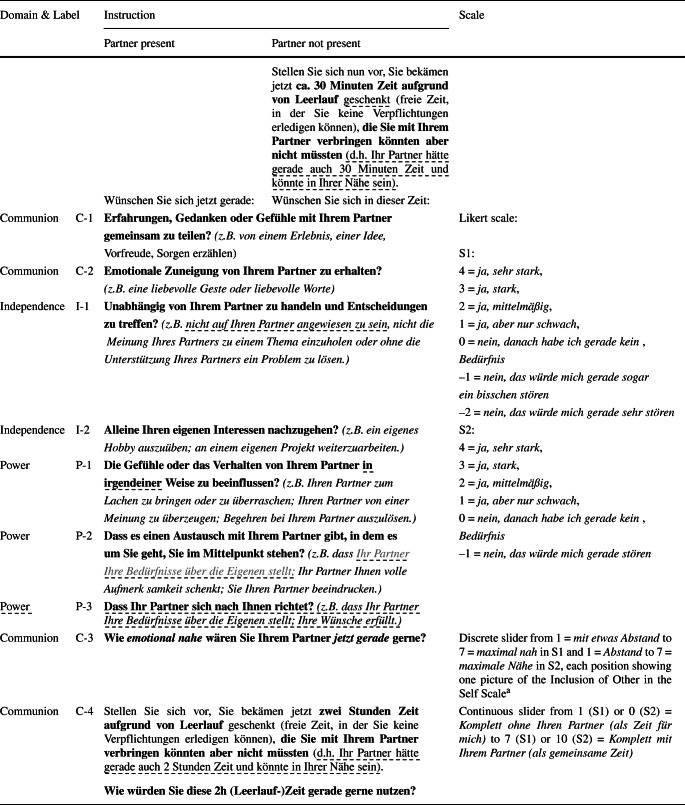
Original experience sampling items for the assessment of motivation

Dashed underlined black text was not part of the items in S1, dashed underlined gray text was only part of the item in S1

^a^ Aron, Aron, & Aron, (1992). We consider item C4 as bipolar item that covers both the communal and agentic domain with regard to independence. In the current analyses, we only use the item as a communion item. These items can be reused under a CC-BY4.0 license

**Table 10:**
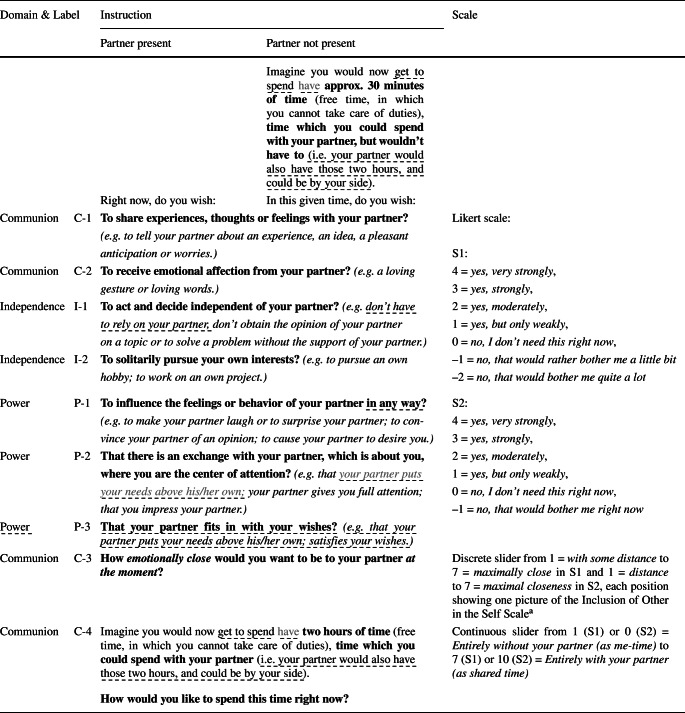
English translation of experience sampling items for the assessment of motivation

S1 = Sample 1, S2 = Sample 2. Dashed underlined black text was not part of the items in S1, dashed underlined gray text was only part of the item in S1

^a^ Aron, Aron, and Smollan (1992). We consider item C4 as bipolar item that covers both the communal and agentic domain with regard to independence. In the current analyses, we only use the item as a communion item. These items can be reused under a CC-BY4.0 license

## Footnotes

1 We note that this practice makes the scale score sample-dependent, which is undesirable if the absolute value of a score should be interpreted. Alternatively, items could be rescaled to the same response scale.

2 Specifically, the five surveys per day were pseudo-randomly distributed across the day. Start and end time could to some extent be personalized and some time spans of each day could be blocked in S2 because participants knew that they would not be able to answer in these periods.

3 Note that Shrout and Lane (2012) do not include $\sigma ^{2}_{\text {TIME}*\text {ITEM}}$ in their Eq. (8), although time is considered random for this equation.

4 If the maximum number of days and moments is inserted, instead of the average number of answered moments and days, reliabilities are virtually identical for *R*_*B**C*_ (up to +.003) and *R*_*B**P*_ (S1: +.005, S2: +.003), and slightly larger for *R*_*W**P**D*_ (S1: +.027, S2: +.021). In a previous publication based on S1 (Zygar et al., 2018b), a shorter two-item scale for communal motivation was employed, consisting of items C-1 and C-2 (see Table 9). This more homogenous scale demonstrated the following reliabilities in the larger S2 sample: *R*_*B**C*_ = .58, *R*_*B**P*_ = .97, *R*_*W**P**D*_ = .88, and *R*_*W**P**M*_ = .70.

## References

[CR1] Adolf, J. K., & Fried, E. I. (2019). Ergodicity is sufficient but not necessary for group-to-individual generalizability. *Proceedings of the National Academy of Sciences*, 201818675. 10.1073/pnas.1818675116. 10.1073/pnas.1818675116 PMC6452692 30872469

[CR2] Aron A, Aron EN, Smollan D (1992). Inclusion of Other in the Self Scale and the structure of interpersonal closeness. Journal of Personality and Social Psychology.

[CR3] Arslan, R. C., Walther, M. P., & Tata, C. S. (2019). Formr: A study framework allowing for automated feedback generation and complex longitudinal experience-sampling studies using R. *Behavior Research Methods*. 10.3758/s13428-019-01236-y. 10.3758/s13428-019-01236-y PMC7005096 30937847

[CR4] Bakan D (1966). The duality of human existence: An essay on psychology and religion.

[CR5] Bates D, Mächler M, Bolker B, Walker S (2015). Fitting linear mixed-effects models using lme4. Journal of Statistical Software.

[CR6] Berridge KC (2004). Motivation concepts in behavioral neuroscience. Physiology & Behavior.

[CR7] Bolger N, Laurenceau J-P (2013). Intensive longitudinal methods: An introduction to diary and experience sampling research.

[CR8] Borkenau P, Ostendorf F (1998). The big five as states: How useful is the five-factor model to describe intraindividual variations over time?. Journal of Research in Personality.

[CR9] Brose A, Voelkle MC, Lövdén M, Lindenberger U, Schmiedek F (2015). Differences in the between-person and within-person structures of affect are a matter of degree. European Journal of Personality.

[CR10] Cranford JA, Shrout PE, Iida M, Rafaeli E, Yip T, Bolger N (2006). A procedure for evaluating sensitivity to within-person change: Can mood measures in diary studies detect change reliably?. Personality and Social Psychology Bulletin.

[CR11] Cronbach, L. J., Gleser, G. C., Nanda, H., & Rajaratnam, N. (Eds.) (1972). *The dependability of behavioral measurements: Theory of generalizability for scores and profiles*. New York: Wiley.

[CR12] Dewitte M, Mayer A (2018). Exploring the link between daily relationship quality, sexual desire, and sexual activity in couples. Archives of Sexual Behavior.

[CR13] Fisher AJ, Medaglia JD, Jeronimus BF (2018). Lack of group-to-individual generalizability is a threat to human subjects research. Proceedings of the National Academy of Sciences.

[CR14] Fleeson W (2001). Toward a structure- and process-integrated view of personality: Traits as density distributions of states. Journal of Personality and Social Psychology.

[CR15] Grice JW, Jackson BJ, McDaniel BL (2006). Bridging the Idiographic-Nomothetic divide: A Follow-up study. Journal of Personality.

[CR16] Hagemeyer B, Neberich W, Asendorpf JB, Neyer FJ (2013). (In)Congruence of implicit and explicit communal motives predicts the quality and stability of couple relationships. Journal of Personality.

[CR17] Hagemeyer B, Neyer FJ (2012). Assessing implicit motivational orientations in couple relationships: The Partner-Related Agency and Communion Test (PACT). Psychological Assessment.

[CR18] Hagemeyer B, Neyer FJ, Neberich W, Asendorpf JB (2013). The ABC of social desires: Affiliation, being alone, and closeness to partner. European Journal of Personality.

[CR19] Hagemeyer B, Schönbrodt FD, Neyer FJ, Neberich W, Asendorpf JB (2015). When ‘together’ means ‘too close’: Agency motives and relationship functioning in coresident and living-apart-together couples. Journal of Personality and Social Psychology.

[CR20] Heckhausen, J., & Heckhausen, H. (Eds.) (2018). *Motivation and Action (3rd ed.). Springer International Publishing. Retrieved April 26, 2019, from* https://www.springer.com/de/book/9783319650937.

[CR21] Hofmann W, Finkel EJ, Fitzsimons GM (2015). Close relationships and self-regulation: How relationship satisfaction facilitates momentary goal pursuit. Journal of Personality and Social Psychology.

[CR22] Hofmann W, Vohs KD, Baumeister RF (2012). What people desire, feel conflicted about, and try to resist in everyday life. Psychological Science.

[CR23] Horstmann KT, Ziegler M (2020). Assessing personality states: What to consider when constructing personality state measures. European Journal of Personality.

[CR24] Impett EA, Gable SL, Peplau LA (2005). Giving up and giving in: The costs and benefits of daily sacrifice in intimate relationships. Journal of Personality and Social Psychology.

[CR25] Karney B, Bradbury T (1995). The longitudinal course of marital quality and stability: A review of theory, method, and mesearch. Psychological Bulletin.

[CR26] Kenny DA, Kashy DA, Cook WL (2006). Dyadic data analysis.

[CR27] Kievit, R., Frankenhuis, W. E., Waldorp, L., & Borsboom, D. (2013). Simpson’s paradox in psychological science: A practical guide. *Frontiers in Psychology*, *4*. 10.3389/fpsyg.2013.00513. 10.3389/fpsyg.2013.00513 PMC3740239 23964259

[CR28] Kindt S, Vansteenkiste M, Loeys T, Goubert L (2016). Helping motivation and well-being of chronic pain couples: A daily diary study. Pain.

[CR29] Kirtley, O. J., Hiekkaranta, A. P., Kunkels, Y. K., Eisele, G., Verhoeven, D., Van Nierop, M., & Myin-Germeys, I. (2020). The experience sampling method (ESM) item repository. *OSF*. 10.17605/OSF.IO/KG376.

[CR30] Lane SP, Shrout PE (2010). Abstract: Assessing the reliability of Within-Person change over time: A dynamic factor analysis approach. Multivariate Behavioral Research.

[CR31] Laurenceau J-P, Bolger N (2005). Using diary methods to study marital and family processes. Journal of Family Psychology.

[CR32] McClelland DC (1987). Human motivation.

[CR33] Medaglia, J. D., Jeronimus, B. F., & Fisher, A. J. (2019). Reply to Adolf and Fried: Conditional equivalence and imperatives for person-level science. *Proceedings of the National Academy of Sciences*, 201820221. 10.1073/pnas.1820221116. 10.1073/pnas.1820221116 PMC6452707 30872468

[CR34] Molenaar PC (2008). On the implications of the classical ergodic theorems: Analysis of developmental processes has to focus on intra-individual variation. Developmental Psychobiology.

[CR35] Muise A, Impett EA, Desmarais S (2013). Getting it on versus getting it over with: Sexual motivation, desire, and satisfaction in intimate bonds. Personality and Social Psychology Bulletin.

[CR36] Nezlek, J. B. (2016). A practical guide to understanding reliability in studies of within-person variability. *Journal of Research in Personality*, 1–7. 10.1016/j.jrp.2016.06.020.

[CR37] Pusch S, Schönbrodt FD, Zygar-Hoffmann C, Hagemeyer B (2020). Truth and wishful thinking: How interindividual differences in communal motives manifest in momentary partner perceptions. European Journal of Personality.

[CR38] Core Team R (2020). R: A language and environment for statistical computing. manual R Foundation for Statistical Computing.

[CR39] Schoebi D (2008). The coregulation of daily affect in marital relationships. Journal of Family Psychology.

[CR40] Schönbrodt FD, Gerstenberg FXR (2012). An IRT analysis of motive questionnaires: The Unified Motive Scales. Journal of Research in Personality.

[CR41] Schultheiss OC, Brunstein JC (2010). Implicit motives.

[CR42] Schultheiss, O. C., & Köllner, M. G. (2021). Implicit motives. In O. P. John, & R. W. Robins (Eds.) *Handbook of personality psychology: Theory and research (4th ed.)* (pp. 385–410). New York: Guilford Press.

[CR43] Scott, S. B., Sliwinski, M. J., Zawadzki, M., Stawski, R. S., Kim, J., Marcusson-Clavertz, D., ..., Smyth, J. M. (2018). A coordinated analysis of variance in affect in daily life. *Assessment*, 107319111879946. 10.1177/1073191118799460. 10.1177/1073191118799460 PMC6408986 30198310

[CR44] Shavelson RJ, Webb NM (1991). Generalizability theory: A primer.

[CR45] Shrout, P. E., & Lane, S. P. (2012). Psychometrics. In M. R. Mehl, & T. S. Conner (Eds.) *Handbook of research methods for studying daily life*. Retrieved November 23, 2018, from https://www.guilford.com/books/Handbook-of-Research-Methods-for-Studying-Daily-Life/Mehl-Conner/9781462513055/contents (p. New York): Guilford Press.

[CR46] Suessenbach F, Loughnan S, Schönbrodt FD, Moore AB (2019). The dominance, prestige, and leadership account of social power motives. European Journal of Personality.

[CR47] Winter DG (1994). Manual for scoring motive imagery in running text (4th edn.).

[CR48] Zygar C, Hagemeyer B, Pusch S, Schönbrodt FD (2018). From motive dispositions to states to outcomes: An intensive experience sampling study on communal motivational dynamics in couples. European Journal of Personality.

[CR49] Zygar, C., Hagemeyer, B., Pusch, S., & Schönbrodt, F. D. (2018b). *From motive dispositions to states to outcomes: Research data of an intensive experience sampling study on communal motivational dynamics in couples (Version 2.1.0) [data and documentation]*. Trier: Psychologisches Datenarchiv PsychData des Leibniz-Zentrums für Psychologische Information und Dokumentation ZPID. Retrieved June 13, 2018, from 10.5160/psychdata.zrce16dy99_v20100.

[CR50] Zygar-Hoffmann, C., Hagemeyer, B., Pusch, S., & Schönbrodt, F. D. (2020). *Eine große Längsschnittstudie zu Motivation Verhalten und Zufriedenheit von Paaren: Forschungsdaten einer vierwöchigen Experience-Sampling-Studie mit einer Vor- Nach- und einjährigen Follow-up-Befragung. [A large longitudinal study on motivation, behavior and satisfaction in couples. Research data from a four-week experience sampling study with a pre-, post-, and one-year follow-up assessment.]* Trier: Psychologisches Datenarchiv PsychData des Leibniz-Institut für Psychologie ZPID. 10.5160/psychdata.zrce18mo99.

[CR51] Zygar-Hoffmann, C., Pusch, S., Hagemeyer, B., & Schönbrodt, F. D. (2020). Motivated behavior in intimate relationships: Comparing the predictive value of motivational variables. *Social Psychological Bulletin*, *15*. 10.32872/spb.2873.

[CR52] Zygar-Hoffmann C, Schönbrodt FD (2020). Recalling experiences: Looking at momentary, retrospective and global assessments of relationship satisfaction. Collabra: Psychology.

